# Predicting Areas with High Concentration of the Long-Term Uninsured and Their Association with Emergency Department Usage by Uninsured Patients in South Carolina

**DOI:** 10.3390/healthcare10050771

**Published:** 2022-04-21

**Authors:** Khoa Truong, Julie Summey Bedi, Lingling Zhang, Brooke Draghi, Lu Shi

**Affiliations:** 1 Department of Public Health Sciences, Clemson University, Clemson, SC 29634, USA; bdraghi@g.clemson.edu (B.D.); lus@clemson.edu (L.S.); 2 Scale Strategic Solutions, Cincinnati, OH 45246, USA; jsb@scalestrategicsolutions.com; 3 College of Nursing and Health Sciences, University of Massachusetts, Boston, MA 02125, USA; lingling.zhang@umb.edu

**Keywords:** long-term uninsured, Emergency Department, Zip Code Tabulation Area, health policy, health disparities

## Abstract

Background: To predict areas with a high concentration of long-term uninsured (LTU) and Emergency Department (ED) usage by uninsured patients in South Carolina. Methods: American Community Survey data was used to predict the concentration of LTU at the ZIP Code Tabulation Area (ZCTA) level. In a multivariate regression model, the LTU concentration was then modeled to predict ED visits by uninsured patients. ED data came from the restricted South Carolina Patient Encounter data with patients’ billing zip codes. A simulation was conducted to predict changes in the ED visit numbers and rates by uninsured patients if the LTU concentration was reduced to a lower level. Results: Overall, there was a positive relationship between ED visit rates by the uninsured patients and areas with higher concentrations of LTU. Our simulation model predicted that if the LTU concentration for each ZCTA was reduced to the lowest quintile, the ED visit rates by the uninsured would decrease significantly. The greatest reduction in the number of ED visits by the uninsured over a two-year period was for the following primary diagnoses: abdominal pain (15,751 visits), cellulitis and abscess (11,260 visits) and diseases for the teeth and supporting structures (10,525 visits). Conclusions: The provision of primary healthcare services to the LTU could help cut back inappropriate uses of ED resources and healthcare costs.

## 1. Introduction

Reducing health disparities and improving the health and well-being of all Americans is one of the key objectives of Healthy People 2020 and Healthy People 2030 [1,2]. However, health insurance is a necessary condition for most Americans to receive healthcare services, and many uninsured individuals face financial barriers to obtaining adequate coverage [2].

Uninsured individuals were more likely to forgo or delay necessary preventive and medical health services for fear of unaffordable medical bills, resulting in worsened health conditions, increased unnecessary morbidity and premature death [3]. It is also documented that uninsured individuals used the Emergency Department (ED) as their ultimate source of healthcare [4]. According to the 2011 National Hospital Ambulatory Medical Care Survey, uninsured adults were more likely to visit the ED because they had no other place to go at the time of the last visit (61.6%) compared with adults having private insurance (38.9%) or those with public health plan coverage (48.5%) [5]. 

While it might be a coping strategy in a desperate situation, the ED is an expensive and inappropriate place to obtain care. Many ED visits are not considered to be emergencies and could be prevented or better handled in different healthcare settings. For instance, most dental visits to the ED result in temporary treatment with painkillers or antibiotics for symptom relief and no treatment for the underlying diseases [6,7,8]. It is estimated that 56% of ED visits, worth $38 billion in annual healthcare spending, are considered avoidable [9,10]. The COVID-19 pandemic has impacted ED usage across the United States. During the early months of the pandemic, an estimated 42% decrease in total ED visits nationwide was reported. Since a proportion of uninsured individuals mainly obtain healthcare services from EDs, health status and healthcare access may be significantly affected by the ongoing pandemic [11].

The pattern of ED use may vary by the length of time without health insurance. A core subset of the uninsured population is referred to as the long-term uninsured (LTU) [12]. LTU adults (uninsured ≥1 year) were much more likely than the short-term uninsured to have not had a routine checkup in the last 2 years (42.8% vs. 22.3%) [13]. Findings from the Medical Expenditure Panel Survey highlighted the importance of considering the length of time without coverage in evaluating preventive service use of the uninsured population [14]. 

Using access measures including not only medical care but also dental care and prescription drugs, Abdus indicated that the LTU were much less likely to have a usual source of care, compared to those who were uninsured for a shorter time [15]. Although the classification of LTU may vary in different studies, it is generally no less than one year without coverage. Based on 2018 National Health Interview Survey data, an estimated 13.3% of adults (ages 18–64) were uninsured at the time of the interview, while approximately 7.9% of adults (ages 18–64) were categorized as LTU [16].

To the best of our knowledge, no study has looked at the aggregate relationship between areas with higher concentration of LTU and ED visits by the uninsured. In fact, there is no estimate of LTU at smaller geographical units, such as ZIP codes or even counties. We aimed to contribute the literature by using publicly available data to predict concentrations of the LTU and exploring a plausible relationship between the predicted measure of LTU and ED usage by the uninsured at the zip code level in South Carolina. 

We undertook the following tasks. First, various sources of data were used in a statistical model to predict and categorize the concentrations of the LTU. Second, ED patient-level data were used to calculate the numbers and rates of ED visits regarding the top reasons by uninsured patients. Third, in a multivariate regression model at the ZIP code level, the LTU concentration level was modeled to predict ED usage by the uninsured. Lastly, we estimated changes in ED usage by the uninsured in response to simulated changes in the LTU concentration level.

## 2. Materials and Methods

### 2.1. Data

The data used to construct the dependent variable was from the ED component of the South Carolina (SC) Patient Encounter Database. The ED component contained a universe of ED visits in SC from January 2012 through December 2013 (N = 4,076,200 visits). The episode-level data contained patient demographics, diagnoses, procedures performed and the charges incurred. 

With a focus on the uninsured population, we included ED visits only by the uninsured, which included self-pay or indigent/charitable organization as opposed to any other primary payer (Medicare, Medicaid, commercial insurance, Worker’s Compensation, other government or HMO). Other inclusion criteria included patients ages 18–64 with a valid SC zip code from their billing address. After applying all inclusion criteria, 1,062,418 ED visits remained in the analytical sample. 

We used the terms ZIP Codes and ZIP Code Tabulation Area (ZCTA) interchangeably but noting that there is a slight difference between ZIP code and ZIP Code Tabulation Area (ZCTA). “Unique” ZIP codes for specific large entity, such as a military base, a hospital or a university are excluded from this study. “Standard” ZIP codes with a “regular” population, defined by the Census Bureau as ZCTAs, served as the unit of analysis in our analysis. Aggregate analyses at the ZCTA level have been performed elsewhere [17,18,19]. The study protocol and methods were approved by the Institutional Review Board of Clemson University who also determined that the use of data in this manuscript does not involve human subjects and was thus not subject to IRB review.

### 2.2. Dependent Variable—ED Usage by the Uninsured

The top reasons for ED visits were identified using ED Minor Diagnostic Categories (MDCs)-subcategories of Major Diagnostic Categories [20]. A frequency tabulation was generated for the ED minor diagnostic category assigned to each ED visit for all patients in the sample. 

Out of 1,062,418 ED visits by the uninsured, the top 15 most frequent ED minor diagnostic categories were chosen for this analysis and will henceforth be referred to as “conditions”: Abdominal pain (N = 53,034); chest pain (N = 47,742); cellulitis and abscess (N = 40,839); diseases of the teeth and supporting structures (N = 39,380); contusions with intact skin surfaces (N = 38,228); other symptoms, signs and ill-defined conditions (N = 32,378); other dorsopathies (N = 32,352); headache (N = 24,971); other sprains and strains of the back (N = 24,327); urinary tract infection, site not specified (N = 24,207); other injuries (N = 23,776); derangements and other unspecified joint disorders (N = 21,146); acute bronchitis and bronchiolitis (N = 20,508); springs and strains of the neck (N = 18,585); and other rheumatism excluding back (N = 16,927). 

**Figure 1:**
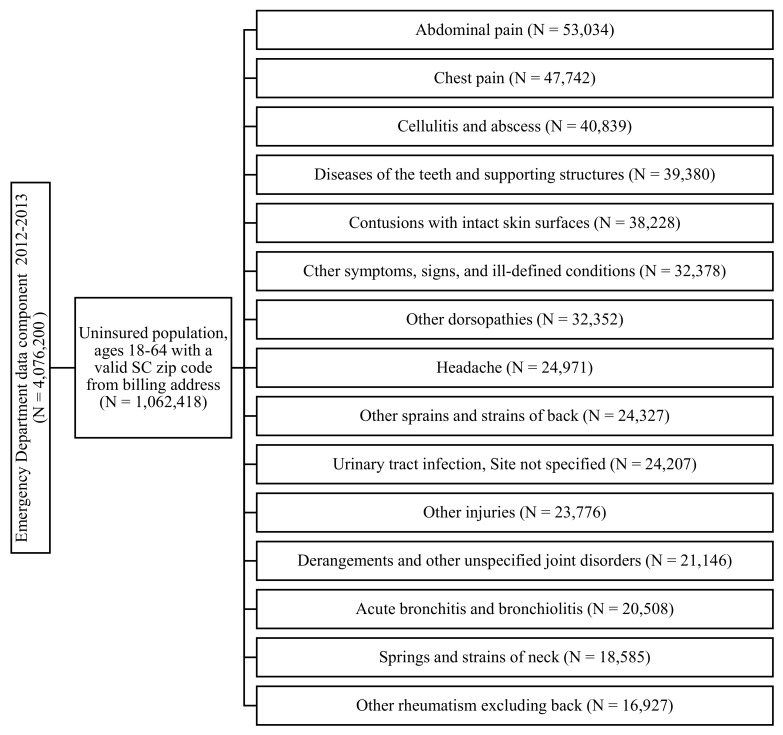
Data management flow chart.

Figure 1 provides a summary of how the data samples for each of the top 15 conditions were created from original ED data component with a focus on the uninsured population. The dependent variable was defined as the ED visit rate by the uninsured for a specific condition per 1000 persons at the ZCTA level. The rate was calculated by taking the total number of ED visits in 2012 and 2013 for a specific condition divided by the population of the ZCTA aged 18–64 years in 2012 and 2013 and multiplied by 1000. The population data was obtained from the US Census Bureau [21]; therefore, the ED visit rates were also for those two years.

### 2.3. Key Independent Variable

After excluding 32 smaller ZCTAs due to missing data, we included 392 ZCTAs covering more than 95% of SC population. To the best of our knowledge, there is no survey that has estimated the percentage of the long-term uninsured population at the ZCTA level. Existing statistics only provide estimates the of uninsured population, which is quite different from the long-term uninsured. We attempted to predict the concentration of the long-term uninsured by categorizing these 392 ZCTAs into five quintiles with Q1 denoted as the lowest and Q5 as the highest level of LTU concentration through multiple steps. First, guided by a literature review, key characteristics of LTU individuals were identified: uninsured status, minority, low education level, unemployment, non-family households and living in poverty [22,23,24,25,26,27,28]. 

Second, data from the American Community Survey [29] were used to measure those characteristics: percentage uninsured aged 18–64 years (average rate 2008–2012), percentage Hispanic, percentage with less than high school education, percentage unemployed aged 16 and older, percentage of non-family households and percentage population living below 200% of poverty level. Third, we estimated a propensity score for the LTU of the ZCTAs using a logistic regression model. 

The resulting predicted propensity scores for each ZCTA, ranging from zero to one (the greater the value, the more likely to contain LTU individuals) were divided into five quintiles, with the top 20% (Q5) containing the ZCTAs with the highest predicted LTU concentration and the bottom 20% (Q1) containing the ZCTAs with the lowest predicted LTU concentration. Using ArcGIS (Environmental Systems Research Institute, Redlands, CA, USA), a map of geographical distribution of the LTU by ZCTAs was created. Research to predict the long-term uninsured has been performed elsewhere [23]. The key independent variables are therefore a set of dummy variables for LTU concentrations.

### 2.4. Statistical Analysis

A multivariable linear regression (ordinary least squares) model was used to examine the relationship between the ED visit rate for each of the 15 top ED visits by uninsured persons and the constructed measure of LTU. The robust variance estimator was applied to correct the conventional standard error of the estimates for the cases in which the regression error is unequal or serially correlated. The dependent variable was ED visit rate by the uninsured. The key independent variables were the LTU quintile dummy variables Q2–Q5, with Q1 (lowest LTU concentration) serving as the reference group. 

We hypothesized that ZCTA with higher LTU concentrations would be positively associated with higher rate of ED visits by the uninsured. Therefore, we expect the sign for each coefficient of Q2–Q5 to be positive. Each estimated coefficient can be interpreted as the increase (if positive coefficient) or decrease (if negative coefficient) in the two-year ED visit rate for a specific condition per 1000 persons for ZCTAs classified as LTU quintile Qi when compared to LTU Q1.

Other explanatory variables (at the ZCTA level) also included information from the Uniform Data System (UDS) Mapper [30]: the percentage of adults who are obese (2009–2012), percentage of adults who have delayed or not sought care due to high cost (2009–2012) and percentage of adults with no usual source of care (2006–2011). Two additional explanatory variables, merged at the county level, were added to the model for diseases of the teeth and supporting structures: the dentist ratio per thousand persons in the population (2013) from County Health Ranking [31] and percentage of adults with no dental visit in the past year (2008, 2010 and 2012) from the UDS Mapper.

### 2.5. Simulation

From a policy perspective, it would be interesting to understand how a reduction of the LTU would reduce the number of ED visits given their relationship. Coefficients estimated from the regression model do not provide an easy-to-understand answer to the previous question. We therefore built a simple simulation model to translate the regression outputs into practical and meaningful results. In our simulation, the following steps were conducted. 

First, we assumed that policy was to be implemented to increase insurance coverage for the LTU. Second, the level of LTU in all ZCTA were of the same level of the LTU in Q1. Third, after each regression (i.e., for each dependent variable—the ED visit rate by the uninsured for a specific condition) was run, we replaced the data of the actual LTU concentration in Q2–Q4 with the LTU of Q1. 

We then predicted the dependent variable outcome with the previous estimated coefficients and newly added data. In doing so, this simulation yielded the ED visit rates for the 15 conditions if all ZCTAs had the lowest LTU concentration, while the other characteristics were held constant. Last, we calculated the number and difference of visits between the status quo (i.e., without changes of the LTU levels) and the simulated rates. Statistical analysis was conducted in Stata 14 [32].

## 3. Results

**Figure 2:**
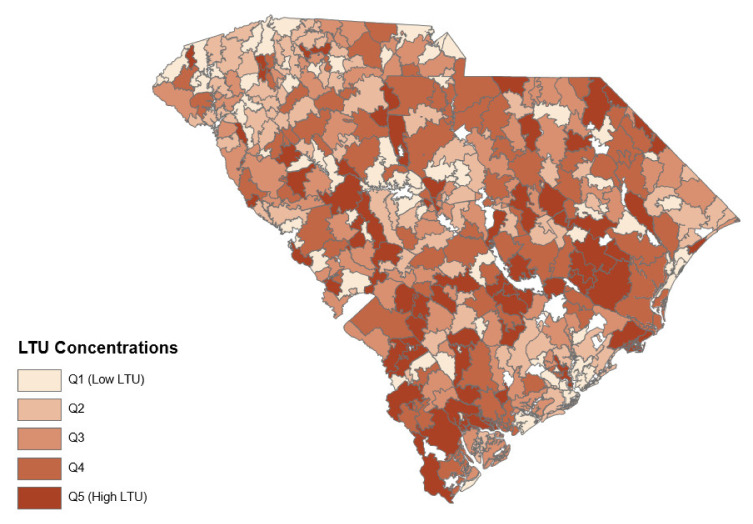
Geographical distribution of the Long-Term Uninsured (LTU) in South Carolina by Zip Code Tabulation Area.

Figure 2 presents the geographical distribution of the LTU by the ZCTAs. The darkest color highlights ZCTAs with the highest concentration of the LTU (Q5) while the lightest color indicates ZCTAs with the least concentration of the LTU (Q1). ZCTAs predicted as Q5 tend to focus more in the Lowcountry region (the South) of South Carolina. The Upstate region (Northwest) seems to fare better but does have pockets of Q5 as well.

Table 1 presents descriptive statistics for the SC ZCTAs and SC Patient Encounter data. The top panel provides the key characteristics of the population for five LTU quintiles. The percentage of the white population decreased consistently from Q1 to Q5. In contrast, the percentage of African Americans, percentage of the population with less than high school education, percentage of the population living below the poverty level, unemployment rate, percentage of adults who have delayed care or not sought care due to high cost, percentage of adults with no usual source of care, and percentage of obese adults all steadily increased from Q1 to Q5. The bottom panel of Table 1 presents the top ED visits by condition per 1000 population. 

The top conditions, in order from highest to lowest, were abdominal pain; chest pain; cellulitis and abscess; diseases of the teeth and supporting structures; other symptoms, signs and ill-defined conditions; other dorsopathies; headache; other sprains and strains of the back; urinary tract infection, site not specified; other injuries; derangements and other unspecified joint disorders; acute bronchitis and bronchiolitis; sprains and strains of the neck; and other rheumatism. It is interesting to observe that the rates for each condition increased consistently from Q1 to Q5 as well.

Table 2 presents the results of the regression model for abdominal pain. All four coefficients of Q2–Q5 have the expected sign (positive). Compared to Q1, LTU Q2 (*p* = 0.02), LTU Q3 (*p* < 0.001), LTU Q4 (*p* = 0.05) and LTU Q5 (*p* = 0.05) are significantly associated with a higher rate of abdominal pain ED visits by the uninsured. We also see that the percentage white (*p* = 0.004), percentage black or African American (*p* = 0.02) and percentage of the population with less than high school education (*p* = 0.01) were statistically significant covariates.

Table 3 presents a summary of the regression results for the other top 14 conditions where only the coefficients for the key independent variables (Q2–Q5) are provided. Compared to LTU Q1, LTU Q2 is a statistically significant predictor for four conditions, LTU Q3 is a significant predictor for eight conditions, LTU Q4 is a significant predictor for five conditions, and LTU Q5 is a significant predictor for two conditions. All of these statistically significant coefficients have the expected sign (positive). The condition that has all four significant coefficients is “other dorsopathies”. 

The condition that has three significant coefficients is “other rheumatism”. The conditions that has two significant coefficients are “cellulitis and abscess”, “diseases of the teeth and supporting structures”, “headache”, “other sprains and strains of the back” and “other injuries”. Conditions that do not have any significant coefficients are “chest pain”, “contusions with intact skin surfaces”, “urinary tract infection, site not specified”, “derangements and other unspecified joint disorders” and “sprains and strains of the neck”. 

With regards to the other socio-demographic independent variables (estimated outputs not shown in Table 3), similar results were observed. The percentage of African American, percentage of population with less than high school education and percentage of adults who have delayed or not sought care due to high cost were found to be statistically significant at the 5% level in most of the models. The percentage living below the poverty level, unemployment rate for population aged 16 years and over, percentage of adults with no usual source of care and percentage of obese adults were also found to be statistically significant in some other top conditions.

Table 4 presents results from our simulation model. If the LTU concentration for each ZCTA is reduced to Q1, the mean ED visit rates by the uninsured for each condition decreases. By applying LTU Q1 to each ZCTA, the greatest reductions in visits by the uninsured are for abdominal pain (15,751 visits), cellulitis and abscess (11,260 visits) and diseases for the teeth and supporting structures (10,525 visits). Examining overlapping confidence intervals, we see that the difference in the status quo and simulated mean ED visit rates are statistically significant for ten of the fifteen conditions.

## 4. Discussion

Measures of the LTU in small geographical areas is virtually non-existent in the current literature. Our constructed measure of the LTU at the ZIP code level (based on American Community Survey data) is a strong predictor of ED usage by the uninsured (from a different data source—SC Patient Encounter). The descriptive statistics clearly shows that the ED visit rates for each of the top 15 conditions increased steadily from the lowest to highest LTU concentration. In the multivariable regressions, 53 out of 60 coefficients (88.34%) had the expected sign (positive), and 24 of these coefficients were statistically significant. 

We expected that higher LTU quintiles would have significantly different ED visit rates compared with lower LTU quintiles. Surprisingly, LTU Q5 was significant in only three conditions. One possible explanation is that Q5 ZCTAs are typically located in more remote location and are the most disadvantaged (evidenced by the descriptive statistics of Table 1). Preventative healthcare services and ED are far less an option for the uninsured population in Q5. 

One factor that contributes to inappropriate use of the ED is physical access. Q3 contains ZCTAs that tend to be located in urban settings, and this could explain why it is more strongly associated with ED usage by the uninsured. After all, how strong the constructed measure of the LTU hinges on the argument that longer periods without insurance coverage would lead to greater use of the ED.

The 2014 Affordable Care Act (ACA) provisions that significantly addressed the uninsured population included the expansion of Medicaid eligibility, individual mandate and creation of health insurance exchanges. A previous study found a substantial decrease in ED visits by uninsured patients (ages 18–64 years) following the insurance expansion [33]. However, a differing study identified a small decrease in ED visits by uninsured patients without comorbidities but a 15% increase in ED visits by uninsured individuals living in high-poverty ZIP codes in Maryland [34].

A positive association between ED usage by the uninsured and the LTU is supported by the existing literature [34,35]. A major reason for usage of ED services by the uninsured is lack of primary care access [7,36,37,38]. In 2015, approximately 25% of all adult Americans did not have a source of primary care and a lack of insurance contributes to less primary care services received by the uninsured [39]. While a typical procedure costs more in the ED than in a clinic [5,40,41], this problem cannot be simply viewed from a cost-saving perspective. 

People used EDs as their primary source of care for a variety of reasons [42]. Previous research has identified potential ways to lessen uninsured ED usage by increasing primary care visits. One study found that small monetary incentives offered to low-income uninsured adults encouraged these individuals to seek primary care, thus, decreasing non-emergent ED visits [43]. A study found that increasing the geographic density of Federally Qualified Health Centers (FQHCs) resulted in a significant reduction in ED visits by uninsured adults [44].

Limitations of our study should be recognized. First, information available from the Patient Encounter ED data does not allow us to determine a visit as urgent or non-urgent. It is even harder to determine a visit as avoidable or non-avoidable. Therefore, conclusions regarding whether an ED visit is necessary are not possible. One only can say at the aggregate level whether the use of the ED is appropriate for a condition, such as diseases of the teeth and supporting structures. Second, our unit of analysis is at episodes/visits rather than patients, i.e., “ED frequent flyers” were not considered. Either measure (patient or episode) has its own pros and cons. Last, the analysis assumed that self-pay patients were uninsured, which might not always be true.

### Health Equity Implications

Our study uniquely measures the LTU for a small geographical area. The results of our analysis have practical implications for healthcare providers to plan services in disadvantaged locations (a list of ZCTAs for each quintile is available upon request). Inappropriate ED usage could increase the cost of healthcare for all as well as adversely affect the quality of care for ED patients with life-threatening conditions. There have been challenges of the ACA’s Marketplaces [45], and LTU is likely to be a persistent problem. 

One specific recommendation is to focus on the top health conditions that brought uninsured individuals to an ED. The provision of low-cost services, such as mobile clinics [46], could be a practical solution to help these patients.

## Figures and Tables

**Table 1:** Descriptive statistics of the ZCTAs and the top reasons for ED visit rates by uninsured patients across LTU quintiles, mean (standard deviation).

	Quintile 1 (N = 80)	Quintile 2 (N = 78)	Quintile 3 (N = 79)	Quintile 4 (N = 77)	Quintile 5 (N = 78)
% white	81 (16)	72 (20)	66 (20)	58 (22)	49 (24)
% black or African American	18 (16)	26 (20)	32 (20)	40 (22)	48 (25)
% with <high school education	13 (9)	17 (7)	19 (6)	23 (8)	26 (6)
% below poverty level	12 (8)	16 (6)	18 (6)	22 (7)	29 (7)
Unemployment rate (age 16+)	7.7 (3.7)	10.7 (4.3)	13.1 (4.6)	13.8 (4.5)	18.5 (7.6)
% of adults who have delayed care or not sought care due to high cost	17 (3)	18 (2)	19 (3)	19 (04)	20 (4)
% of adults with no usual source of care	18 (3)	19 (4)	18 (4)	18 (4)	18 (5)
% obese adults	29 (5)	30 (5)	32 (5)	33 (6)	34 (7)
% of adults with no dental visit in the past year	34 (7)	36 (5)	39 (6)	39 (7)	41 (7)
Dentist ratio per 1000 population	0.43 (0.22)	0.43 (0.21)	0.35 (0.18)	0.35 (0.21)	0.35 (0.19)
Prevalence of ED Visit By Condition Per 1000 Population					
Abdominal pain	6.1 (4.5)	8.8 (5.0)	10.8 (6.5)	10.1 (5.9)	10.8 (7.2)
Chest pain	6.3 (6.7)	8.2 (3.8)	9.5 (6.0)	10.4 (4.8)	10.9 (5.5)
Cellulitis and abscess	4.8 (4.5)	6.7 (3.6)	9.9 (15.7)	8.7 (4.0)	8.2 (4.8)
Diseases of the teeth and supporting structures	4.5 (4.8)	6.0 (4.0)	8.1 (4.7)	8.0 (4.6)	6.9 (4.4)
Other symptoms, signs and ill-defined conditions	4.2 (4.0)	5.2 (2.6)	6.2 (3.3)	6.1 (3.3)	7.0 (4.2)
Other dorsopathies	3.9 (3.7)	5.9 (3.2)	6.2 (3.2)	6.6 (3.7)	6.9 (3.5)
Headache	2.5 (2.1)	5.1 (7.1)	4.9 (2.4)	5.6 (3.1)	5.8 (3.5)
Other sprains and strains of back	2.7 (2.4)	4.4 (3.1)	5.4 (3.5)	5.3 (3.1)	5.5 (3.2)
Urinary tract infection, site not specified	3.3 (5.5)	3.8 (2.5)	4.7 (2.5)	5.7 (3.3)	5.5 (3.5)
Other injuries	3.1 (3.5)	4.4 (2.6)	5.2 (4.8)	4.5 (1.9)	5.1 (2.7)
Derangements and other unspecified joint disorders	2.8 (3.1)	3.3 (1.8)	4.3 (4.9)	4.0 (2.5)	4.5 (2.8)
Acute bronchitis and bronchiolitis	2.5 (3.1)	3.2 (2.3)	4.6 (4.5)	3.9 (2.7)	4.0 (2.8)
Sprains and strains of the neck	2.4 (2.8)	2.9 (1.8)	4.1 (2.6)	4.0 (2.6)	4.5 (3.2)
Other rheumatism	1.8 (1.6)	2.6 (1.4)	3.3 (1.8)	3.4 (2.0)	3.6 (2.4)

Note: LTU = Long-term uninsured (Quintile 1 = Lowest LTU Concentration and Quintile 5 = Highest LTU Concentration).

**Table 2:** Regression Results for Abdominal Pain (ED Visit Rates as the Dependent Variable), South Carolina 2012–2013.

Explanatory Variables	Coefficient	95% CI
LTU Quintile		
1 (reference)		
2	1.96 **	[0.35, 3.57]
3	3.40 ***	[1.56, 5.23]
4	2.40 **	[−0.01, 4.80]
5	2.74 **	[0.02, 5.46]
% white	0.29 ***	[0.09, 0.49]
% black or African American	0.25 **	[0.05, 0.45]
% of population with less than high school education	18.46 ***	[4.64, 32.28]
% below poverty level	−0.01	[−0.16, 0.13]
Unemployment rate for population age 16 years and over	0.08	[−0.09, 0.26]
% of adults who have delayed or not sought care due to high cost	15.80	[−10.68, 42.28]
% of adults with no usual source of care	13.92	[−6.10, 33.94]
% obese adults	1.39	[−14.74, 17.52]

Notes: CI, confidence interval; LTU, Long-term uninsured (1 = Lowest LTU and 5 = Highest LTU); Boldface indicates statistical significance (** *p* < 0.05 and *** *p* < 0.01). Prob > F = 0.0000; R-squared = 0.3209; Adj R-squared = 0.2706.

**Table 3:** Regression Results for 14 Other Top Conditions (ED Visit Rates as the Dependent Variable), South Carolina 2012–2013.

LTU Quintile					
	1 (Reference Group)	2	3	4	5
	Coefficient [95% CI]				
Chest pain		0.8 [−1.3, 2.9]	1.4 [−1.1, 3.8]	1.9 [−0.9, 4.7]	1.5 [−2.2, 5.2]
Cellulitis and abscess		1.1 [−0.5, 2.7]	3.7 *** [1.2, 6.1]	2.4 * [−0.05, 4.8]	1.7 [−1.3, 4.7]
Diseases of the teeth and supporting structures		0.99 [−0.4, 2.4]	2.8 *** [1.2, 4.4]	2.7 *** [0.8, 4.6]	1.3 [−1.2, 3.8]
Contusions with intact skin surfaces		−0.03 [−1.4, 1.3]	1.2 [−0.4, 2.8]	0.4 [−1.7, 2.6]	−1.2 [−3.9, 1.4]
Other symptoms, signs and ill-defined conditions		0.5 [−0.7, 1.7]	1.1 * [−0.2, 2.5]	0.7 [−1.1, 2.5]	1.4 [−1.0, 3.7]
Other dorsopathies		1.63 *** [0.4, 2.8]	1.7 ** [0.3, 3.1]	1.9 ** [0.3, 3.5]	2.2 ** [0.3, 4.1]
Headache		1.7 *** [0.5, 2.9]	1.0 [−0.4, 2.3]	1.4 * [−0.2, 2.9]	0.6 [−1.9, 3.1]
Other sprains and strains of the back		0.9 ** [0.0, 1.7]	1.3 *** [0.3, 2.3]	0.7 [−0.6, 1.9]	0.2 [−1.6, 2.0]
Urinary tract infection, site not specified		−0.08 [−1.5, 1.3]	0.5 [−1.1, 2.1]	1.1 [−0.7, 2.8]	0.5 [−1.6, 2.5]
Other injuries		0.8 * [−0.1, 1.7]	1.3 ** [0.2, 2.4]	0.5 [−0.5, 1.6]	1.0 [−0.3, 2.3]
Derangements and other unspecified joint disorders		−0.04 [−1.0, 0.9]	0.6 [−0.6, 1.9]	−0.01 [−1.4, 1.4]	0.02 [−1.8, 1.8]
Acute bronchitis and bronchiolitis		0.2 [−0.6, 1.1]	1.3 *** [0.3, 2.3]	0.5 [−0.6, 1.6]	0.4 [−0.9, 1.7]
Sprains and strains of the neck		−0.3 [−1.0, 0.5]	0.4 [−0.4, 1.3]	0.0 [−1.0, 1.0]	−0.1 [−1.4, 1.2]
Other rheumatism excluding back		0.4 [−0.1, 0.9]	1.0 *** [0.3, 1.6]	0.8 ** [0.1, 1.6]	0.8 * [−0.1, 1.8]

Notes: CI, confidence interval; LTU, Long-term uninsured (1 = Lowest LTU and 5 = Highest LTU); Boldface indicates statistical significance (* *p* < 0.10, ** *p* < 0.05 and *** *p* < 0.01).

**Table 4:** Simulated Changes in ED Visits in Response to Changes in the LTU for Quintile 1.

	Status Quo ^1^		Simulated ^2^		Difference	
	Mean ED Visit Rate by the Uninsured per 1000 Population ^3^	Number of ED Visits	Simulated Mean ED Visit Rate per 1000 Population ^2^	Number of ED Visits	Change in Actual and Simulated ED Visit Rate per 1000 Population ^2^	Change in Number of Actual and Simulated ED Visits
Abdominal pain	9.3 [8.7, 9.9]	53,034	7.2 [7.0, 7.4]	37,283	−2.1	−15,751
Chest pain	9.1 [8.5, 9.6]	47,742	8.0 [7.8, 8.2]	42,593	−1.1	−5149
Cellulitis and abscess	7.7 [6.9, 8.5]	40,839	5.9 [5.6, 6.2]	29,579	−1.8	−11,260
Diseases of the teeth and supporting structures	6.7 [6.2, 7.2]	39,380	5.1 [5.0, 5.3]	28,855	−1.6	−10,525
Contusions with intact skin surfaces	7.5 [7.1, 8.0]	38,228	7.4 [7.2, 7.7]	37,892	−0.1	−336
Other symptoms, signs and ill-defined conditions	5.7 [5.3, 6.1]	32,378	5.0 [4.9, 5.1]	26,403	−0.7	−5975
Other dorsopathies	5.9 [5.5, 6.2]	32,352	4.4 [4.3, 4.5]	24,502	−1.5	−7850
Headache	4.8 [4.3, 5.2]	24,971	3.8 [3.7, 4.0]	19,945	−1.0	−5026
Other sprains and strains of the back	4.7 [4.3, 5.0]	24,327	4.1 [3.9, 4.2]	20,199	−0.6	−4128
Urinary tract infection, site not specified	4.6 [4.2, 5.0]	24,207	4.2 [4.1, 4.3]	22,357	−0.4	−1850
Other injuries	4.4 [4.1, 4.8]	23,776	3.7 [3.6, 3.8]	19,781	−0.7	−3995
Derangements and other unspecified joint disorders	3.8 [3.5, 4.1]	21,146	3.7 [3.6, 3.8]	19,151	−0.1	−1995
Acute bronchitis and bronchiolitis	3.6 [3.3, 4.0]	20,508	3.1 [3.0, 3.3]	16,359	−0.5	−4149
Sprains and strains of the neck	3.6 [3.3, 3.9]	18,585	3.6 [3.4, 3.7]	18,345	0.0	−240
Other rheumatism excluding back	2.9 [2.7, 3.1]	16,927	2.3 [2.3, 2.4]	12,740	−0.6	−4187

^1^ The status quo is the current stage of the world; ^2^ The simulated world is all ZCTAs have the same level of LTU as that of Q1 (lowest LTU concentration) and all other characteristics are held constant; ^3^ The population refers to the population aged 18–64 years of each SC ZCTA for two years of data (2012 and 2013).

## Data Availability

There are two sources of data for this manuscript: Patient information came from a restricted dataset that was provided by South Carolina Revenue and Fiscal Affairs Office–Health and Demographics after being approved by the Data Oversight Council. Zip-code level data is publicly available from the Census Bureau.
